# Care coordination for chronic and complex health conditions: An experienced based co-design study engaging consumer and clinician groups for service improvement

**DOI:** 10.1371/journal.pone.0224380

**Published:** 2019-10-31

**Authors:** Liza Heslop, Kathryn Cranwell, Trish Burton

**Affiliations:** 1 Institute for Sport and Health, College of Health and Biomedicine, Victoria University, Melbourne, Victoria, Australia; 2 Community Integration, Allied Health & Service Planning, Western Health, Sunshine, Victoria, Australia; 3 Nursing and Midwifery, James Cook University, Cairns, Queensland, Australia; Aga Khan University, KENYA

## Abstract

**Background:**

Evidence shows that engaging consumers and clinicians in development of health services creates a more responsive, integrated service that better meets the needs of consumers and the community of practice it serves. Further, consumer and clinician participation in service development processes can boost confidence and motivation levels in organisational employees and help foster clinical accountability.

**Objective:**

To see where consumers’ care experiences could be improved by better understanding where care coordination organisational systems needed improvement.

**Methods:**

Experienced based co-design informed an investigation of consumer and clinician experiences of a care coordination service and involved the sharing of those experiences across service employees in a series of iterative and feedback loops over eighteen months (July 2012-January 2014). Formal participants included care coordination clinicians (n = 13) and consumers. Data from formal participants were collected during September-December 2012, consisting of consumer video-recorded and clinician audio-recorded interviews. Interview transcriptions were analysed to identify service “touch points”, being emotionally significant events related to key service aspects that connect or disconnect consumers and/or clinicians.

**Results:**

Results revealed that consumers highly valued the transdisciplinary skill base of the care coordination workforce, though service improvements were needed for transition support, quality discharge planning and conveying better understandings of care coordination activity both internally and externally.

**Conclusion:**

Incorporating consumer and clinician view-points about their experiences, including the production of a DVD, facilitated conversations across the entire service about care coordination provision and provided a catalyst for design improvement that may otherwise have been difficult to achieve. Some changes to the service were made such as improved client complaints processes, new roles for the care coordination service, and enlisting clinical staff to undertake motivational interviewing training to promote greater consumer self-management capacity. In this study, the user experience was given a platform within a larger healthcare workforce capability development project.

## Introduction

Public health and social services nationally and internationally have noticed an increase in the quantity of older people presenting to health services suffering from long-term, complex multi-morbidity with unique problems, characteristics and needs [1–2]. Problems identified as commonly experienced by consumers with multi-morbidity include fragmentation and poor coordination of care, polypharmacy, mental health difficulties, functional difficulties, reduced quality of life, and treatment burden—all of which lead to increased healthcare utilisation [3].

People who experience long term chronic health conditions frequently find it difficult to navigate complex and unfamiliar healthcare systems and require specialist services. To assist vulnerable populations navigate healthcare systems, maximise their levels of functioning, and quality of life and health status, a care co-ordination service model is recommended [4]. Care co-ordination is a generic term for processes by which healthcare professionals are clinically responsible for monitoring and co-ordinating healthcare options and outcomes for people who experience complex social, environmental, financial and cultural factors impacting on their health. Care coordination arrangements differ from country to country [5]. Generally, the goals of care co-ordination include: enhancing consumers’ quality of life; management and co-ordination of healthcare services to encourage a continuous service experience for consumers; the provision of quality interventions and; decreasing the fragmentation of care where multiple treatment modalities are involved [6]. In previous research, Heslop, Power and Cranwell [7] described care coordination workforce functions that were associated with delivering person-centred care for at-risk groups revealing clinician direct care functions by domain viz. access, assessment, providing consultation, arranging care, contracting, treatment and preparation/administration.

The aim of this study was to gather in-depth understandings from consumers and clinicians about their experiences of care coordination service provision, and to discern and share “touch points” for whole of service learnings with the intent of improving how care coordination services are delivered. Both this study and the previous research [7] were part of the Health Workforce Australia, Aged Care Workforce Reform Program (complex care coordination in the community)—an initiative of the Commonwealth of Australia supporting reforms over an 18-month period, July 2012 to January 2014.

## Materials and methods

### Design, setting and methodology

Using experienced based co-design (EBCD), as developed by the King’s Fund and currently available as a tool kit from the Point of Care Foundation [8], the EBCD process was undertaken over an eighteen-month period (July 2012-January 2014) within a care coordination service provided by a Local Healthcare Network (LHN) situated in the outer western metropolitan region of Melbourne, Victoria, Australia. The region has a high proportion of culturally and linguistically diverse (CALD) communities where English may not be the first language; and where the population comprises a higher burden of disease than national averages, a high proportion of socio-economic disadvantage, and low levels of health literacy [9]. The organisational view was that empowering consumers was key to service improvement for at-risk populations and was a way forward to encourage social inclusion initiatives that may reduce healthcare access inequities.

This study was underpinned by the EBCD approach, where methods are known to enhance engagement with local communities [10]. Further, the methods resonated with the service objectives to strengthen existing practices and improve the capacity of care coordinators to deliver coordinated, comprehensive and accessible person-centered care to consumers with complex and chronic healthcare needs. EBCD is a form of participatory action research that fundamentally involves people working collaboratively to develop practical service improvements, and to enhance skills and knowledge in people and organisations by exploring and sharing subjective experiences of consumers, carers, and healthcare personnel [11]. In this study, consumers are “clients” and “carers”. Six stages of EBCD have been described [12]. We adapted these, as outlined in Table 1, to suit available resources, the consumer cohort, and the healthcare setting.

**Table 1:** Study design: EBCD stages, processes and feedback loops.

Stages in experience-based co-design	Processes and feedback loops
Engage clinical staff from the care coordination service	A service-wide workshop was held to present study information resulting in enlistment of clinical staff representatives from each care coordination service program, and the formation of an EBCD steering group to champion the overall EBCD process and assist with recruitment, data collection, analysis and co-design activities
Recruit consumer group from the care coordination service	Provide study information to the consumer group; obtain consumer group consent to partake in video-recorded interviews, and co-design activities
Conduct clinician interviews	Obtain clinician consent; conduct in-depth clinician audio-taped interviews and transcribe verbatim
Analysis of in-depth clinician interviews	Reading and re-reading of clinician interview transcripts by research team; identification of touchpoints as well as soliciting feedback from clinicians at several EBCD steering group meetings
Video record interviews with consumer participants	Consent obtained from consumer participant group
Review consumer video-recorded interviews, identify touch points and use in feedback loops with EBCD steering group to make final selections for a DVD production.	The mapping of clinician and client themes were discussed at the EBCD steering group, and it was agreed that around ten key quotes from both consumer and clinician staff experiences—illustrative of touch points—would be selected for the DVD. The DVD produced was titled: ‘Someone was there for me’. The DVD was shown to clinician and consumer groups and at an entire staff service forum. Later in the EBCD process, the DVD was used again with the consumer group at a final joint event
Joint clinician/consumer feedback event	In April 2013, a joint event was held. In attendance were 18 consumers from the care coordination services, KC and LH, 7 consumers from the formal consumer participant cohort, and members of the EBCD steering group. The purpose of the meeting was to show the DVD and seek comment from those present
Implementation of service improvement	Prioritisation of initiatives and co-design groups established to deliver on service improvement

The study was granted ethical approval on the 09 July 2012, project number QA2012.53 from the Low Risk Human Research Ethics Panel Office for Research, Western Centre for Health Research and Education Sunshine Hospital, Furlong Rd. St Albans VIC 3021.

### Data collection

As EBCD processes adopt an entire service approach, many informal participants were involved at different points in time. For instance, key to driving EBCD processes internally was the appointment of clinical champions and an EBCD steering group. Formal participants are described below.

#### Participant descriptions and recruitment

The care co-ordination clinical staff members who participated in the study were employed in Western Health’s Care Coordination Service which, at the time of this study, included five programs known as: Immediate Response Service (IRS); Hospital Admission Risk Program (HARP); Aged Care Assessment Service (ACAS); Post-Acute Care Service (PAC); and Community Based Transitional Care Program (TCP). The professional disciplines represented by the care co-ordination staff included Registered Nurses, Occupational Therapists, Physiotherapists, Pharmacist, Social Workers and Drug and Alcohol clinicians. At the time of the study, the clinician equivalent full time of the service was ninety-four. Thirteen service clinicians were recruited by KC and the EBCD steering group and gave written consent as formal participants.

Consumers who participated were all current or recent users of the care coordination service. A senior care coordinator was appointed to coordinate the identification of potential participants from the five care coordination programs with the assistance of clinicians of the service. Potential consumer names were forwarded to the senior clinician care coordinator, who made contact with them to explain the study purposes and processes. Consumer selection was purposive. CALD consumers participation was supported through the availability of interpreters. Twenty-five consumers were contacted with fifteen agreeing to be interviewed. Consumers excluded were those with current Victorian Civil Administrative Tribunal applications, in a complaint’s process, or if cognitively impaired. Fourteen consumers meeting the inclusion criteria provided written consent to participate and were interviewed in their home. One consumer was interviewed at the facility following an outpatient appointment. The consumer age range was 59–95 years. Of those who agreed to participate, eight were from CALD backgrounds. All five western region Local Government Areas were represented, as were all care coordination programs. Interpreters were offered but declined. Participating consumers were diagnosed as suffering from conditions such as chronic obstructive pulmonary disease, Parkinson’s disease, diabetes, Alzheimer’s, dementia, complex orthopaedic conditions (upper limb amputee, and fractures with reduced mobility), post-polio, and cardiac conditions. The break down according to service was PAC-1; TCP-2; IRS-3; ACAS-6; HARP-4. Interviews lasted approximately forty-five minutes to one hour. Reimbursement of transport and provision for respite costs were provided.

#### Instruments

Interviews with both clinicians and consumers occurred during September-December 2012 with questions focused on dimensions of experience. Interview questions were guided by and adapted from the principles of person-centred care as identified by the Picker Institute: Information and knowledge, care planning and flow of treatment, fragmentation of holistic care, collaboration, consistency, connection.

Clinicians were interviewed about their experiences concerning the strengths and weaknesses of continuity and coordination, how to improve the service and what they considered were critical elements of the program [S1 Text]. These interviews were digitally recorded, and subsequently transcribed verbatim and de-identified for analysis.

Consumer interview questions [S2 Text] requested information regarding consumer’s experience of ease of access to treatment, care co-ordination, the value of health education and information provided, and the impact of the support given by staff. Equally the interviews requested information concerning how the consumer experienced the involvement of family and friends in their treatment, the consumer’s relationship with staff, the value of treatment within their own environment and their experience of treatment continuity.

### Data analysis

Consumer and clinician narratives were examined for ‘touch points’. Touch points in EBCD represent critical moments or events and are considered expressions of subjective and emotional experiences [13]. Baldly stated, touch points indicate where emotional and cognitive connections are, or need to be established. Touch points, in this study, denote personal and crucial memories that shape experience of a service, and where consumers and clinicians connect or disconnect with a care coordination service, and where their experience of the service is formed. The analysis team comprised researchers with inputs from care coordination clinicians and the EBCD steering group.

The transcriptions of clinicians’ interviews were read and reread by the researchers to identify touch points. Each consumer video recording was presented to the EBCD steering group over four meetings for note taking. Recordings were poised to facilitate collective discussion and to confer on touch points. The touch points selected for inclusion in the video were agreed upon by all team members with key consumer quotes marked for extraction and production of a short film to depict the consumer experience. The final product was a DVD lasting approximately 10 minutes that included, also, contextual information, client footage illustrating touch points and staff quotes to support consumer footage.

As part of the EBCD process, the DVD was subsequently viewed and discussed by 65% (62 out of 94) of care coordination clinicians in several groups. It served as a communication aid to facilitate client involvement and facilitated conversations between staff about the client story. Staff were able to identify how important their role was to consumers as well as identify opportunities for service improvement.

Finally, the DVD was used in a joint event as part of the EBCD process. At this event, clinicians and consumers watched the DVD which acted as a tool to support participants to better understand the consumer experience, other clinicians’ experiences and identify opportunities for service improvement. Items for service improvement were prioritised, and co-design groups of consumers and clinicians were formed to deliver these improvements [S3 Text].

#### Qualitative research reporting standard

This study follows the COREQ checklist for qualitative standards [S4 Text]. Of note is that EBCD derives from ethnographic traditions. Within that tradition, the relationship between participants and researchers acknowledges that values intrude upon and are unavoidable in any investigation [14]. This means that our writing strategies incorporated the value orientation of the researchers as gained from personal experience. Nevertheless, the limits of interpreting texts are acknowledged in this study. In constructing a textual account, the researcher appeals to particular categories, which are always articulated from the researcher’s experiential location. Researchers speak from experience and the authorities gained from that experience in a world that is known to them. In this study, personal experiences were drawn upon to explicate what consumers highly valued and to discern opportunities to make improvements. Thus, the epistemological foundations of qualitative studies in the ethnographic tradition differ considerably from traditional qualitative methods and may not be reproducible.

## Results

### Staff audio-recorded interviews

Touch points were identified according to two main areas: 1) Critical moments for clinicians and what they perceived to be critical moments for consumers; 2) Where services failed and what could be done to improve. The summarised points itemised in Table 2 are drawn from the data analysis of clinician interviews [S5 Text] and summarised based on Picker principles.

**Table 2:** Clinician touch points.

**Critical moments for clinicians**		
Picker principle	Touch point	Illustrative clinician text
Information and knowledge	Managing unreal expectations; Not clear cut what we do; Lack of program knowledge from providers and consumers	*There is a limitation about what difference we can make for people*, *sometimes what the person needs is not actually available and so you have to adjust what you can do*. *Sometimes people’s expectations are that you will have a magic wand and you don’t have a magic wand [laughter]* #40
Collaboration	Team functioning–sharing of experiences and knowledge	*Everyone seems ready to share knowledge and also impart knowledge*. *If someone’s looking a bit lost or not certain about something*, *I’ve never felt any sense of anyone being afraid to ask a question or ask a bit of advice* #9
Connections	Enhancing access to vulnerable groups and providing links across healthcare systems; Advocacy and empowerment	*On the positive side*, *for them [consumers]*, *… they’re usually really clear if someone’s actually listening; who [else has] got the time to sit down for one and a half*, *two hours at that first visit and get all that information from them*. *A lot of them*, *they feel like they’re heard*. *They’re acknowledged and respected and heard* #33
**What clinicians perceived to be critical moments for clients**		
Picker principle theme	Touch point	Illustrative clinician text
Fragmentation of holistic care	Waiting times; Overwhelming and confusing/complex system	*… frustrating is the waiting times for some of the services that we’re approving people for #32* *When they [consumers] are in the ED waiting*, *sometimes it is nice to touch base with them*, *provide family with a chair*, *introduce who you are*, *give that familiar face again and explain what’s going on in the emergency department*. *And that they’ve not been neglected*, *and they will be seen … if you address those parts*, *you can make it really smooth but if those bits are neglected*, *I think some patients can become quite overwhelmed*, *frustrated*, *feel as if they’re being neglected* #44
Care planning and flow of treatment	Client no idea how to navigate system; Client slips through the cracks	*And if people haven't gone home with a letter from the hospital about the plan e*.*g*. *referred to outpatients*, *the GP*, *post-acute care and the council*. *If you don't know the plan*, *it's very hard for [consumers] people to take control* #47
Connections	Managing distress at hospital triage; Immediacy of connection with client (first encounter); Being listened to over a period of time	… *really important that the person who rings on intake feels that they’ve been listened to–the client*, *the carer has been listened to*. *And you might make some suggestions … and half the time the people just feel that they’ve managed to pour out their story to the right person who is then going to get someone to come* … #41

### Consumer video-recorded interviews

Table 3 provides a summary of touch points from consumer video-recorded interviews. We adapted a structure from the Picker principles of person-centered care to guide the presentation of touch points.

**Table 3:** Touch points derived from consumer video-recorded interviews.

**Aspects of care coordination highly valued**	
Information and knowledge	Clinicians expert knowledge of client and care needs; Provided advice on *‘things I didn’t know I needed’*
Collaboration	Equal say; Clients as partners; Advocate for client—such as liaison with GP
Care planning and flow of treatment	Coordination of external services; *‘I would have been totally lost’*
Connections	Encouragement; Establishing rapport; Accommodating; ‘*Treats us as individuals’; ‘Understands our concerns’*
Consistency	Consistency of staff important
**Areas for improvement—care coordination**	
Information and knowledge	Written information was not considered useful; Access: ‘*We didn’t realise we could access these services…’* Information sharing: For example, ‘*GP was not advised or provided a discharge summary’*
Collaboration	Passivity—lack of involvement in decision making; ‘*Informed of the care plan’* rather than active participants; Directed and ‘*advised’* rather than actively involved in decisions; Views ‘*dismissed’*; Not listened to
Fragmentation of holistic care	Feeling of abandonment on cessation; ‘*I wish they were still coming’*; Siloed approaches to care delivery; Poor information sharing across the continuum of care; Lack of coordination of care between services
Care planning and flow of treatment	‘*Someone needs to be in charge’*; Inpatient experiences ‘traumatic’; Transition from inpatient to home and the importance of being able to meet clinicians who provide follow up care/services

The summaries of data analysis sessions for the DVD production are available on request. Consumers valued establishing connections and building rapport with clinicians and respected their expert knowledge. Consumers placed importance on clinicians who had understood their history and care needs, had “done their homework” and were aware of what had happened before in the care journey. Areas noted by consumers needing improvement were issues around fragmentation of holistic care due to poor coordination in some areas, which led to gaps in information sharing with consumers, and in sharing of information between services. Although the large majority of consumers reported feeling a sense of inclusion in their care planning, there were others who alluded to being ‘informed’ rather than being active participants.

### Final joint event

Clinicians received feedback on how valuable the service was perceived by consumers at the final consumer-clinician joint event. At this event the DVD, “Someone was there for me”, was reviewed. One consumer stated: *I’m a carer and someone looking in*. *One of the most important things I see is the medical staff see you in hospital at the bad stage*, *and then you’re sent home … when home you can’t go back to hospital … you can’t go to the doctor every five minutes and you put it off*, *as it’s so physically hard to do … so you get depressed and give up …*. *[the service] installs that confidence in people*, *gives them the will to go on because they get personalised treatment–very person [centred] and that is what you need … because they see people at this stage better equipped than a doctor or hospital and see the next step … doctors don’t give or can’t give in-depth time … but team people know what worked for this one and that one …*. *I saw that work several times… acutely terminal and a hell of a lot better twelve months later … because of back up team and the input of knowledge they know from working with people on the ground*. Clinicians perceived that celebrating successes alongside identifying service opportunities to be a balanced perspective.

The consumer group tended to be overly positive about the service and eliciting consumer touch points about negative experiences of care posed a challenge for the codesign model. For example, the facilitator tried desperately to elicit shortfalls of the service from the consumer group but still a consumer stated: *Not much to be faulted … your service brings trust and confidence…*. *then they [consumers] can feel empowered as you are the backup … from [consumer] perspective*, *so extremely important … doctors bogged down in medical speak …* Consumers often remarked that information about the service should be widely available; but at the time of the study the care coordination service was not considered internally to be an “open service” but rather one to target unique needs of a particular population group. Thus, the LHN care coordination service as a resource was not boundless and was not marketed too broadly.

### Key changes

As part of the EBCD process, several potential changes (Table 4) to the practice of care coordination were, and are continuing to be implemented to improve the quality of service provision.

**Table 4:** Service changes made resulting from co-design.

**Access**	Marketing of care coordination service to local and inpatient services to support early referral was undertaken by implementing a strategy to increase the profile of the service within the organisation which was supported by the public affairs team within the LHN.
**Workforce development**	Implementation of competency-based training and education to support development of care coordination trans-professional skills which were highly valued by consumers
**Client feedback**	Increase opportunities for informal consumer feedback through establishment of a clear feedback process and routine provision of a “how to give feedback” brochure produced by the LHN for consumers
**Client transition**	Implementation of strategies to increase client self-efficacy and self-management so clients are discharged with feelings of “empowerment” rather than abandonment. Staff training in motivational interviewing and employment of a support worker to enhance client transition between health sector interfaces.

## Discussion

### Strengths

Actual examples of EBCD implementation with respect to vulnerable groups is generally lacking in practice [15]. A strength of this study was harnessing the time and valuable skills of clinical champions and members of the EBCD steering group which contributed to enhancing consumer and clinician participation and the overall success of the process. Additionally, we achieved widespread service engagement in learnings through feedback loops and the sharing of subjective experiences.

### Limitations and challenges

EBCD upholds connection of consumers with service improvement and is somewhat dependent on their empowerment and engagement. Putting consumer experiences as a knowledge source for service improvement to the forefront of the EBCD process is somewhat tied, nevertheless, to relational dynamics between clinicians and consumers. The achievement of the consumer story must be considered as relational and somewhat shaped between the interactions of the EBCD steering group, clinician participants and the consumer group. In this dynamic, meanings are potentially shaped within a shared orientation between researchers, clinicians and participants. Despite this limitation, we made every effort to ensure consumer experiences were recognized, and not overlooked as a knowledge source for redesign in a complex healthcare context, and for informing workforce development in the larger Health Workforce Australia project. Within that effort we held a final event with consumers and clinicians to strengthen robust dialogues and to build upon highly valued opportunities to prioritise work for co-design that were raised in the process.

We experienced considerable difficulties establishing co-design groups given the consumer population, nevertheless clinical champions continue to make regular contact with some members of the consumer group as part of ongoing efforts to facilitate and sustain uptake of strategies at individual, community and organisational levels.

There were, additionally, challenges raised by clinicians about how to best prioritise access. The care coordination service comprised five different programs and clinician knowledge about each program may have limited their ability to make appropriate referrals and improve consumer access. At EBCD steering group meetings, it was identified that it may not be possible for consumers to differentiate care experiences specifically provided by care coordination services so, as part of the consumer story, inpatient experiences were included but with a focus on care coordination.

A review of EBCD studies conducted in 2016 noted that limited detail was provided at the implementation and evaluation stages [16]. We found EBCD to be a time-consuming and resource-intensive process. We managed the processes within a complex set of cultural and institutional interactions; notwithstanding it was our first experience with it. A major limitation was that we were unable to undertake a formal evaluation of whether the EBCD process led to sustainable improvements in the care coordination service. Still, unproven effects could be the development of a constructive organisational culture and climate on access to care coordination, and development of clinicians’ abilities.

## Conclusion

The EBCD process was an important catalyst scaffolding a care coordination workforce culture that values consumer engagement and the development of constructive organisational cultures. Alternative strategies for sustainable consumer engagement in service improvement, maintaining workforce-related practice changes, and improvements to service following this EBCD study continue to be ongoing challenges. The resource intensive nature of the EBCD process makes it difficult to embed improvements in everyday practice without dedicated resources, though improvements initiated by this study continue to be explored with the intent of ultimately improving the overall capacity of care coordinators to deliver coordinated and comprehensive person-centred care to consumers with complex and chronic care needs.

## Supporting information

S1 Text Script and interview schedule for clinician interviews. (DOCX)

S2 Text Consumer interview schedule. (DOCX)

S3 Text Summary of final joint meeting. (DOCX)

S4 Text COREQ. (DOCX)

S5 Text Data curation clinician interviews. (DOCX)
