# Supplementary material for: Care coordination for chronic and complex health conditions: An experienced based co-design study engaging consumer and clinician groups for service improvement
Source: PLoS One. 2019 Oct 31;14(10):e0224380. doi: 10.1371/journal.pone.0224380 (PMC6822704; doi:10.1371/journal.pone.0224380)
Supplement: S2 Text — (DOCX) [file pone.0224380.s002.docx]

**S2: Consumer interview schedule**

**Introduction**

Introductions, which service interview staff are from, time frame, confidentiality, breaks

Explanation of EBCD project

Consumer/carer & clinicians working to redesign services

Video record of interview for short edited film, with permission

View footage & consent prior to use, all participation voluntary

**Tell me about your journey so far**

| **Access to Care** | How did you come to be seen by X service?  Tell me about your first contact  What challenges with contacting or setting up appointments?  What did you know about X service prior to this? |
| --- | --- |
| **Physical Comfort** | Where did the contact with the X service take place (home, In ED, ACF other)?  If other, can you describe the physical environment and if it felt comfortable  What was it like having X service coming to your home? |
| **Coordination & Integration of Care** | What happened after the first contact from X service?  How did staff ensure that your previous history was taken into account during this contact?  How did key people and services involved in your care work together? |
| **Information, Communication, & Education** | Tell me about how the X service staff communicated with you.  What information did you receive about your health problem and your care?  We are aiming for the **right** information at the **right** time, can you tell me about what it was like for you?  Did you have follow-up contact?  What did you find useful in the written information that was provided to you? |
| **Emotional Support & Alleviation of Fear & Anxiety** | This sounds like it was a stressful time for you, tell me about what it was like?  During your contact with X service was there an opportunity to talk about the stress you experienced?  What supports were offered to you in coping with the stress and other emotions at this time and afterwards?  What other supports did you use?  What do you think would have helped? |
| **Respect for Patients Values, Preferences and Expressed Needs** | Tell me about how staff were involved in your care, their attitudes, manner, communication?  Tell me about your relationships with staff, did you feel part of the team?  Each individual’s situation is different, tell me about how that was for you in your contact with X service, was this recognised?  How much of a say did you have?  How did staff accommodate your personal beliefs, preferences or needs? |
| **Involvement of Family & friends** | How were your family & friends involved in your care?  Would you have liked more or less involvement?  If more, how could you see friends & family becoming more involved? |
| **Continuity & Transition** | Tell me about your follow up after your involvement with X Service  Was your GP aware of the X services involvement?  How much information did your GP receive about your contact with X service?  Any further follow up that would have helped you? |

**Concluding Comments**

Overall what were the most important points in your experience with X service- the moments of truth?

Are there any key points we should focus on in the redesign of X service?

What was the best part and part of most concern of your experience?

Based on your first-hand experience, if you were looking to design and improve the service for patients of the X service where would you begin, if you were setting up from scratch

(Physical environment, process, staff behaviour, attitudes)

**THANKYOU**
