# Supplementary material for: Care coordination for chronic and complex health conditions: An experienced based co-design study engaging consumer and clinician groups for service improvement
Source: PLoS One. 2019 Oct 31;14(10):e0224380. doi: 10.1371/journal.pone.0224380 (PMC6822704; doi:10.1371/journal.pone.0224380)
Supplement: S3 Text — (DOCX) [file pone.0224380.s003.docx]

**S3: Summary of final joint meeting with consumer groups and EBCD working group held at Western Health April 2013**

**Purpose of the meeting**

1. Show video to consumer group.
2. Seek comment from consumer group
3. Summarise comments from consumer group
4. Show video again
5. Collate next round of comments
6. Summarise commentary from the consumer group and note main touch points

**Meeting participants in attendance**

18 consumers (includes carers) from the services, LH (note taker) and KC and 7 research participants from the formal consumer groups

*Names changed in these meeting notes to ensure anonymity

**Introduction**

The facilitator commenced with introductions and explained the EBCD model of working with service users, and the need to get in-depth input about how redesign is working. It was emphasised that the EBCD approach aims to gather unique viewpoints. The value of feedback about how the services are working based upon users’ experiences was also emphasises. Our goal is to improve services and inform and educate staff. As an EBCD working group, we have been working together for some time. The principle of ‘touch points’ is very important to us as these are the critical areas where we want feedback. During the EBCD process, we recruited consumers with different age range and cultural background, and we covered council areas across the West. To complement this, we interviewed13 staff about their experiences.

**Show of video (11 minutes)**

**Initial reactions from consumers after viewing of film**

“Sounds like everyone interviewed had very positive feedback I learnt now about contact with aged care to get mum respite, but I did not realise how many services you provided”.

“I had problems with Parkinson’s where doctor did not help much at all…I thought I had to persevere with what doctors could offer”.

“I was overwhelmed and could not cope… everything up in air ... confusion in hospital, one team then another team … all so busy and none talked to the other. [Clinician] made me feel safe and that someone was there… someone was there…someone cared … was not my own”.

“Medical people so busy with their section and don’t want to hear about all the problems you’ve got… “

“…. well cared for but lack of communication, for example, could not work out which outpatient clinic to attend…”

Facilitator: Does the video reflect the experiences?

“We could have said it [positive comments] even more”.

“I’m a carer and someone looking in. One of the most important things I see is the medical staff see you in hospital at the bad stage and then you sent home… when home you can go back to hospital … you can’t go to the doctor every 5 minutes and you put it off as it’s so physically hard to do… so you get depressed and give up …. Installs that confidence in people, gives them the will to go on because they get personalised treatment – very person [centred] and that is what you need… because they see people at this stage better equipped than a doctor or hospital and see next step … doctors don’t give or can’t give in-depth time … but team people know what worked for this one and that one …. I saw that work several times…acutely terminal and a hell of a lot better 12 months later …because of back up team and the input of knowledge they know from working with people on the ground”.

Facilitator: Commented on people scared and out of control.

“…. they [clinicians] always were there…what will I do with this prednisolone? ... they could advise.”

“…[Clinicians] did make it significant... for example, why you ringing about that…but at one stage…I rang and one of the girls [clinicians] who told me just do this and this till you get to the specialist and they pulled me thru again”.

“…depression was a major factor for me and to have that …support … safety … vitally important to me as well as practice service”.

“…checking appointment; you’ve got this coming up Elizabeth etc…overall holistic….”

“we all know … there is a limit to be delivered due to resources money… this team available so important from financial … gets them [clients] up to keep going instead of falling in a trough rather than to fall to system of expensive care … overall a big improvement in the system”.

“Not just financial, mum preferred to stay at home. Loved all the life in home, not same in nursing homes…”

“Able to be feel so safe”

Facilitator commented about supporting chronic complex conditions in the home

**Rescreening of video**

| **Consumer comments** | **KC and EBCD working group comments** |
| --- | --- |
|  | Communication at various levels and coordination of absolutely everything, but also for clients the awareness of what services to access at what time … longevity of staying at home. |
| To empower you … | The way you spoke about care very powerful … one of the most important things we are doing is listening to you and the way you told those stories was very powerful. |
| Hard on staff as you do have more than one client …very much appreciated … I want to give back … watching it now I don’t think I said enough. | You described the relationship with the staff member as friend – not just about clinical care but that rapport you build with people and trust. |
| Very much … I went to my doctor and he had helped me for ten years. Doctor very sick, very tired, I’m shaking. I’m 68, sent to neurologist … I got Parkinson’s … I can do nothing for you the GP [said]… I went home crying …can’t deal with this pain ... I can’t afford to pay this osteopath … my friend found this help. Through doctor, I knew nothing”. | Incredibly proud to hear – to hear we are doing so well from your perspective … importance of maintaining health and wellbeing…fantastic…we should be a service as positive model for you actually managing your own live rather than medical model which drops you. |
| I felt like I should not call back but then I thought … they are the only ones to ring back. I’m too hard for my GP but always need a specialist … felt secure for a while but when it stopped ... but you [GP] see many patients know you can’t have an ongoing service.  I was so grateful but being told about … accommodation issues, I was given the whole lot…contacts, not just with the condition….  …. hospital speak …very much an advocate for so many areas … as [clinician] said we know who to contact and to navigate | Medical model stops and this model in between. Had those skills but at some point, you needed or knew to call back.  Would you like formalised phone call two months down track…?  Is there any way we could help to prepare you better for that experience where you wanted to give up? |
| Only use [this service] when in crisis … some might call all the time. | Once your case is closed you expressed you felt guilty about recontacting the service … we want to encourage self-management but do not want to leave people stranded … follow up [using] one number where you could call that you could use … possible an indicator we might provide for support …struck me about comments re guilt … |
| Attach a section to the video … a summary …. that this was a concern or that this was highlighted …. a summary of today and the key points of the video… | Initial video just a tool to start discussions … how we can support that for our staff. What can be improved? e.g. follow up…as we continue to work through it, we can add more to video” |
| Seems to be single living on own who need more support that couples … no carers |  |
| And the carer needs a carer too…limits to what family supports can provide |  |

**Final summary of points made on white board**

**Things to continue or do better (taken from board final summary, comments from consumers, facilitator and EBCD working group)**

“Need to explain who you are and what you do and getting across the services … put rest of video in context … reminder needed at the start”.

“We want ideas – we do it well…. we want to do better”

“At the moment tool for making improvement but further down the track a resource”.

“We can come back to that question later but now focus on how we can improve, what you value about your service and what we can improve…the gaps”

"I only heard about it in hospitals”

“GPs don’t know about service…don’t put forward when there is a need for it”.

“? video aims at patient or medicos out there”

“Obligation with better liaison with GPs”

“Team is can do outfit but who … doctors and patient and their families … often about crisis points … stress points for families … how do we know we can access this service when we get home … so hit and miss to access these services”.

“No one there to say get in touch with this service’s ... l’m left with nothing but GP”.

“A lot of the video might be directed to informing”

“I was lucky that hospital did it … often an individual … no automatic exposure to it … a brochure… showing it’s there and available … and how you qualify for it … in house TV system…put on TV system where patient sees it … we recently had friend who had been to a doctor. While waiting in surgery watching film about heart attack … next day able to make phone call … would not have survived if not five minutes later... we have a ‘can do’ team by referrals to systems and treatments … we’ll come and talk to you make a suggestion … but it has to be sick people…”

“Thinks we can think about using the video in those ways”.

“But to improve services…what we can do……after watching the video a second time…. [used as prompt].

“Not much to be faulted … your service brings trust and confidence …. then, they [clients] can feel empowered as you are the backup … from patient perspective so extremely important ... doctors bogged down in medical speak….”

KC…the point of exit of services … real value to inform people about service and this tool to do that…value in other people knowing about it”.

“Most of people in western would not know at least half….”

“Are there any groups we are not reaching …senior citizens, community health, health providers and universities”.

“How people access our services…when you do get in touch great but comes back to if you have the staff….”

Facilitator: “Service was very much based on the staff rapport but what if did not like them or did not connect…what if….”

“Mostly I felt guilty for ringing up ... we never felt uncomfortable able…but could happen as you are vulnerable…”

“Did we have a process about a complaint?”

“There must be some point about our work where you can trust us … yes you are looking holistically”

“It comes across [clinicians] so eager to help in any way she could and wanted to get through asap and nothing was too much trouble”.

“Yes, you felt guilty like patient said and some want to take but nothing too much trouble….as long as they deliver what they say they deliver then that’s only you want….”

“Sounds like we delivered on most dimensions you wanted….”

“It was done to the best…”.

EBCD group member: “Complaints process may not be overt…people tend to not complain…you are happy customers, but we really want to hear problems today”

“Network of linkages ... in my case [patient name] has to deal with network outside of western…

So, to work in this area not just a good health professional but what knowing what exists beyond– finances, housing….”

“More links might help you with so much running round….”

Some things need follow up – preparedness for discharge

Access and marketing (but double-edged sword in the sense the service may have limited capacity). Gap that people don’t know about, I don’t know who referred me in hospital.

Some feedback process like patient advocate rather than formal complaint process…feedback system where things don’t work…

CALD and key target groups such as doctors GPs and in the acute setting marketing and informing services

**Final comments:**

Continuing to support a workforce to do that is multi skilled and putting a value on that…our workforce almost pick up all pieces and a bumpy experience. Putting value on that is a very good and important message … I’m sure at different stages very stressful but your message about flexibility and adaption … so as organisation we need to support our workers. I don’t think how important you are is appropriately considered … they don’t see how easily you can do down-hill and end up worse than you were…

You are here to assist people get their health back … but if you get involved in all these other things – all takes time …

“But part of our job is to give people confidence and skills ... where does my role finish and end….”

**Main issues derived from meeting**

The value and overwhelming gratitude placed on the support derived from clinicians of service by consumers, and the confidence gained by consumers in managing their healthcare which is not provided by GP or any other service.

The holistic approach delivered by clinicians of the service that fill in many gaps for self-care management.

Service is a safety net

The difficulty in finding out about the service and the need to market the service to regional health care providers, especially GPs

Feedback system

Feelings of guilt for over using the service

Emotional support was key. Hospitalisation and GP visits were not adequate for the complexity of consumers health problems and were often a distressing experience as consumers expressed lack of support from traditional services; thus, care coordinators met a service gap to relieve patient distress, and this has influenced consumer perceptions about the value they placed on the skill of the clinicians of the service. Further, emotional support, such as providing access to additional services could play a role in improving consumer healthcare outcomes and has an influence on selfcare management and relieving distress.
