# Supplementary material for: Care coordination for chronic and complex health conditions: An experienced based co-design study engaging consumer and clinician groups for service improvement
Source: PLoS One. 2019 Oct 31;14(10):e0224380. doi: 10.1371/journal.pone.0224380 (PMC6822704; doi:10.1371/journal.pone.0224380)
Supplement: S4 Text — (DOCX) [file pone.0224380.s004.docx]

**S4 Study described according to COREQ Criteria**

Using the COREQ criteria (40) we describe our study in further detail below.

**Domain 1: Research team and reflexivity**

**Personal Characteristics**

**1. Interviewer/facilitator. Which author/s conducted the interview or focus group?**

Clinician interviews: KC and EBCD steering group members

Consumer interviews: KC and EBCD steering group members

Joint event (field notes): A member of the EBCD steering group provided facilitation. LH took detailed notes verbatim using a laptop. KC moderated the group discussion and took further notes.

**2. Credentials. What were the researcher’s credentials? E.g. PhD, MD**

LH: Registered Nurse; BA (social sciences), BApp Sci (AdvNurs), MNurs; PhD (2001)

KC: Registered Occupational therapist, MHlthSc (OccTh), BOccTher, GCertMgt

TB: Registered Nurse, PhD.

**3. Occupation. What was their occupation at the time of the study?**

LH: Associate Professor, Victoria University, Victoria, Australia

KC: Community Services Workforce Lead, Western Health, Victoria, Australia

TB: Senior lecturer, James Cook University, Queensland, Australia

**4. Gender. Was the researcher male or female?**

LH, KC: female. Two EBCD steering group members were male, and the remaining were female.

TB did not participate in data gathering.

**5. Experience and training. What experience or training did the researcher have?**

LH: PhD and Master’s by research in qualitative research. PhD (2011) using ethnographic methods and based insights from the contributions that anthropologists have made to the field of organizational studies. Teaching about qualitative data analysis at Monash University and Victoria University (Australia) over 30 years. Regular in-service training on qualitative methods. Published in qualitative research projects and methodologies.

KC: Leadership experience in workforce development and service improvement.

TB: Senior lecturer in qualitative research. Completed nursing science studies in qualitative research. Supervised qualitative research projects.

**Relationship with participants**

**6. Relationship established. Was a relationship established prior to study commencement?**

EBCD is different from traditional research and draws upon ethnographic methods and participatory action research where researchers are joined with participants in the process. Among participants, a relationship was pre-established with KC and members of the EBCD steering group. This was pivotal for driving the study internally and to assist with contacts according to the sampling strategy and aligns with EBCD methods.

**7. Participant knowledge of the interviewer. What did the participants know about the researcher? e.g. personal goals, reasons for doing the research**All research participants took part in information sessions about the study. All participants received oral and written information about the purpose of the research. KC ensured interviewers presented themselves and their background and role during data collection. Participants were encouraged to ask questions about the researchers.

**8. Interviewer characteristics. What characteristics were reported about the interviewer/facilitator? e.g. Bias, assumptions, reasons and interests in the research topic.**Oversight for data collection was moderated by KC who is experienced with workforce development work, including facilitating meetings and discussions in local communities concerning chronic and complex disease management gender and minority issues. An outcome of this study was to develop clinician skills and to gain further experience with research methods.

LH listened and took detailed notes during some areas of data collection and in review of data.

**Domain 2: study design**

**Theoretical framework**

**9. Methodological orientation and theory. What methodological orientation was stated to underpin the study? e.g. grounded theory, discourse analysis, ethnography, phenomenology, content analysis.**The study was underpinned by an emergent health services improvement methodology known as Experienced Based Co-design (King’s Fund). The King’s Fund methodology was adapted considerably to suit our context and resources, and to achieve a study goal which was to connect patients from an at-risk patient population with care coordination staff. Data gathered by the EBCD method required in-depth description of the consumers’ own unique experiences of the service. To gain in-depth data through surveys can elicit comparisons of consumer experiences over time and can monitor performances, but they do so using predetermined and fixed responses which may not elicit the depth of information required. Semi-structured in-depth interviews on the other hand, as used in this study, elicit rich, detailed and multifaceted information regarding consumers’ and staff experiences. The consumers (carers and patients), and staff stories, or narratives, were audio or video recorded and later transcribed and analysed and used in feedback loops between participants and clinicians of the service. EBCD puts emphasis on the experience and that is what sets EBDC apart from other methodologies. We gave the user experience a platform in a larger Health Workforce Australia aged care workforce project.

**Participant selection**

**10. Sampling. How were participants selected? e.g. purposive, convenience, consecutive, snowball**

Purposive sampling. Please see details in paper.

**11. Method of approach. How were participants approached? e.g. face-to-face, telephone, mail, email**

Consumers: (details in paper)

Clinicians:(details in paper)

**12. Sample size. How many participants were in the study?**

Details in the paper

Several members of the EBCD steering group and clinical champions provided informal feedback and insights during ongoing EBCD meetings which were held monthly during over an 18-month period.

**13. Non-participation. How many people refused to participate or dropped out? Reasons?**

Some attrition occurred with the consumer cohort due to health and social factors.

**Setting**

**14. Setting of data collection. Where was the data collected? e.g. home, clinic, workplace**

Consumers: Could choose place of interview and most were held in the consumer’s home. One consumer came on site at Western Health.

Clinician: All clinican data was collected at the participants’ own workplace, that is the care coordination centre.

**15. Presence of non-participants. Was anyone else present besides the participants and researchers?**

Carers were sometimes present with participants (we consider consumers to be both patients and carers)

**16. Description of sample. What are the important characteristics of the sample? e.g. demographic data, date**

Please see paper.

**Data collection**

**17. Interview guide. Were questions, prompts, guides provided by the authors? Was it pilot tested?**

Interview guides for both consumer and participants groups have been provided as supplementary files. Drafts were discussed by KC and the steering group and modified accordingly. See explanation in paper as to how the questions were devised.

**18. Repeat interviews. Were repeat interviews carried out? If yes, how many?**

No repeat interviews were conducted. The EBCD research process included feedback loops as part of the entire service improvement approach. Meetings were held with service groups and consumer group to present, refine and discuss findings and interpretation.

**19. Audio/visual recording. Did the research use audio or visual recording to collect the data?**

Consumer interviews: Video-recorded

Clinician interviews: Audio recorded

**20. Field notes. Were field notes made during and/or after the interview or focus group?**

Field notes were regularly collected by the EBCD steering group and the researchers both before and after interview processes and on a monthly basis during meetings that occurred during the 18-month EBCD process. These field notes were non-systematic but important to the process. Essentially, the data interpretation feedback loops acted as a catalyst for change that may otherwise have been difficult to achieve.

**21. Duration. What was the duration of the interviews or focus group?**

Please see the paper.

**22. Data saturation. Was data saturation discussed?**

Data saturation is not a relevant concept to EBCD designs. The gathering of ‘touch points’ has been described in the paper.

**23. Transcripts returned. Were transcripts returned to participants for comment and/or correction?**

Transcripts were returned to clinician participants for their comment or correction. Clips extracted from the filmed interviews with consumers were viewed by consumers prior to inclusion in the short film. The final video, as mentioned in the study, was based on touch points derived from both consumer and clinician interviews and these were viewed by consumer groups on two separate occasions for comment. Additionally, clinicians of the EBCD working group also viewed the video as part of the EBCD process.

**Domain 3: Analysis and findings**

**Data analysis**

**24. Number of data coders. How many data coders coded the data?**

Three people did inductive coding, and discussed the structure using a code tree based on Picker Principles

**25. Description of the coding tree. Did authors provide a description of the coding tree?**

Supplementary files show how touch points were achieved

**26. Derivation of themes. Were themes identified in advance or derived from the data?**

In EBCD methods, identifying ‘touch points’ rather than ‘themes’ occurred. Refer to paper for more details.

**27. Software. What software, if applicable, was used to manage the data?**

Microsoft word was the only applicable software. Software was used by technicians to produce the DVD.

**28. Participant checking. Did participants provide feedback on the findings?**

Final results were presented at a joint event including a clinician and consumer groups from the service. This was held Western Health in April, 2013. Summary notes are provided in the supplementary file.

**Reporting**

**29. Quotations presented. Were participant quotations presented to illustrate the themes/findings? Was each quotation identified? e.g. participant number**

Participant numbers are identified in the selected quotes and the details are provided in supplementary files. Due to extensive qualitative data collection, only a summary of ‘touch points’ is provided in the paper.

**30. Data and findings consistent. Was there consistency between the data presented and the findings?**

We believe the data presented as ‘touch points’ is a fair representation of all transcripts. The EBCD process was resource intensive and through a number of team efforts with the EBCD working group, and feedback loops as described, our findings have been consistently presented. We have supplied details of touch points in the supplementary files, and the summary in the manuscript reflects that detail.

**31. Clarity of major themes. Were major themes clearly presented in the findings?**

We tend to use the term ‘touch points’ rather than themes in this EBCD study. Touch points of relevance to this study have been presented that fit with the study aims. There were touch points concerning the clinicians’ views about working as a care coordination, and the value they placed on this type of work such as autonomous roles and workplace flexibility, but these themes were outside the scope of this paper and may be reported in a separate paper.

**32. Clarity of minor themes. Is there a description of diverse cases or discussion of minor themes?**

No minor themes have been reported as the study generated ‘touch points’ rather than themes.
