# Supplementary material for: Care coordination for chronic and complex health conditions: An experienced based co-design study engaging consumer and clinician groups for service improvement
Source: PLoS One. 2019 Oct 31;14(10):e0224380. doi: 10.1371/journal.pone.0224380 (PMC6822704; doi:10.1371/journal.pone.0224380)
Supplement: S5 Text — (DOCX) [file pone.0224380.s005.docx]

**S5: Data curation clinician interviews**

**Selected touch points staff interviews: the detail in these tables (A B & C) informed Table 2 and Table 4 in the manuscript**

Adapted Picker themes to structure analysis, viz Information &

Knowledge; Care Planning & Flow of Treatment; Fragmentation of Holistic Care; Collaboration; Consistency; Connection.

| **Table A: Critical moments for clinicians** | | |
| --- | --- | --- |
| **Picker theme** | **Touch points** | **Clinician verbatim quotes** |
| Information and knowledge | Managing unreal expectations | Unrealistic expectations across the board rather than our specific services is the challenge. #46  We can make recommendations about council services, community packages, district nurse coming in to do x, y and z but at the end of the day, they might be asking for someone to sit in the house with mum 24 hours a day and it doesn’t exist. #46  The naivety of the public about what is actually available is a problem. Their expectations don’t match the reality. #49  There is a limitation about what difference we can make for people, just sometimes what the persons needs is not actually available and so you have to adjust what you can do. Sometimes, people’s expectations are that you will have a magic wand and you don’t have a magic wand [laughter]. #40  And perhaps they haven’t really captured exactly what we do and set false expectations and that’s why it sort of falls apart when we get there. #42  So, they’re grateful of the service and they end up usually being appreciative of it, but to begin with, they feel it’s a very, in some cases, they feel it’s a very poor service; that it’s not what their expectations were. #39 |
| Collaboration | Team functioning- sharing of experiences and knowledge | Everyone seems ready to share knowledge and also impart knowledge. If someone’s looking a bit lost or not certain about something, I’ve never felt any sense of anyone being afraid to ask a question or ask a bit of advice. #9  But I believe that the professional development that we’re doing has – the quality and the scope and also the collaboration, well – there might be a single case study and HARP will present what they would’ve done or what they did for that client and ED will say what they did with that client and ACAS said what component they did with that client. #46 |
| Information and knowledge | Not clear cut what we do: Lack of program knowledge from providers and consumers | I get the impression that the medical staff aren’t always aware of what we do and the service we provide, so there are instances, particularly when there’s rotations and there’s new doctors, that people just get discharged… without the social aspect of why they’re here being considered. #45  Yeah, and working on people in emergency that really need to be here. So that’s one of the difficulties but I always make sure – and I think a lot of the care coordinators do – that they’re sort of jumping up and down in the fishbowl with the new doctors and as they get comfortable. #44  We say often we think a lot of people feel we’re a clerical role, we’re not professionals in any discipline and you can hear them, Why do you need to know about family support if we’re asking you to put in home care? And you can understand that. I didn’t know what PAC did until I came here so that’s probably for me the frustrating part of the job and then the big thing about communication between teams, whether it be us, the ward or us and the other care co-ordinator roles and programs. #48  It’s not just about developing rapport but also getting them [referring to patients] to understand what your role is, which can be a bit vague sometimes because…well, I won’t speak for everyone else, but I don’t think I explain it that well sometimes because it isn’t that clear cut, what we do. #45  I think it’s often difficult for patients to understand why we’re there. I think it hasn’t always been explained to them clearly that a referral has been made, either by their GP or the family, or it has been explained and they’ve forgotten. #32  I think, they think it’s great, but I think most people wouldn’t recognise that its post-acute care because the service is provided by the district nurse or the hospital in the home or the clinic. So, patients don’t understand its post-acute care, I don’t mind that they don’t understand as long as they feel they’re being serviced appropriately, as long as the service is providing what they need; I don’t have an issue with that. #39  Most often the clients that we do have don’t have a lot of exposure to services or people coming into their homes; so, it’s a new thing for them and change is difficult. #42 |
| Connections | Enhancing access to vulnerable groups and providing links across healthcare systems | … think the reason we get that population [vulnerable groups] quite a lot is because we go to them. #2  You get much more success if you meet someone as an inpatient. ‘… Cause quite often, they’ll go, No, no, no, no, no. And then, but once you can get in and talk to them, they usually say, Oh, okay. And I think it’s the way we phrase it, the way we word it. #2  … most of the time we’re seeing people once and yet, over the course of the hour and a half, you’re able to give them information and they are left feeling satisfied. #32  On the positive side, for them, like I said, they’re usually really clear if someone’s actually listening; who’s got the time to sit down for one and a half, two hours at that first visit and get all that information from them. A lot of them, they feel like they’re heard. They’re acknowledged and respected and heard. #33  … whereas if we’ve been able to, the day before they went home, advise them of what was going to be available to them and give them time to digest that information #39  There’s a lot of information to take on at once when we go in to see people, but I think people are always pleased – well, not always pleased cause some people mightn’t like the outcome but I think generally – I think people are happy with – that people are there to help. #41  So there’s a whole lot of stuff around the ED journey but just care coordination and the service that you guys provide, if you had to pick a couple of key moments that affected the clients experience – so an example would be, it could be when you initially see – the first time you see a patient, obviously you need to establish repour with them and get a whole lot of information, so that could be a key moment to them accepting your service. #45  … feeling more assured that they’ve found the right place and they’re moving forward or if we’re at the wrong place, that they’ve been provided with the information and they’re moving forward. #46  ….so, I can say, Look, I got this information from my colleague in the emergency department, she said that you have got, boom, boom, boom, is that all correct? So again, exactly, the client feels more confident that you have actually got that, you’ve got that information, that’s all been transferred, they also don’t get fed up with repeating themselves. #46 |
| Connections | Advocacy and empowerment | Maybe because the GP doesn’t have the information, and the person themselves isn’t very good at telling the GP, explaining everything, so sometimes I do that from a point of view of just trying to get information across. #3  Working with them, trying to keep them empowered, but preparing them of the fact that they don’t actually need help anymore. #2  And it’s about giving them some control. #33  It’s a privilege working with people in the home setting where they are able to be themselves and take a little bit more control. #47  … in my previous role as a clinician, you can go out and you make the decision – you don’t make the decision [in care coordination] – with the input from the client and the carer you can help them reach an outcome for that – for what’s in their best interests and hopefully, taking what they want to do as well. #4 |

| **TABLE B What clinicians perceived to be critical moments for clients** | | |
| --- | --- | --- |
| **Picker theme** | **Touch point** | **Clinician quotes** |
| Fragmentation of holistic care | Waiting times | frustrating is the waiting times for some of the services that we’re approving people for #32  I think this just goes back to the emergency department in general, the waiting time, not really being kept informed as to how much longer the wait might be. #43  Sometimes the waiting to see an outreach service, we are supposed to be fairly – we see people urgently but sometimes because of the fact that a lot of people are part timers, or I don’t know, someone calls in sick or whatever, then they might not get seen for a week or two … #45  So, the fact that it’s a two-year or more waiting list for a package is appalling. #49 |
|  | Overwhelming and confusing/complex system | I think that’s quite confusing because I think there’s a lot of mixed messages being sent to clients. #39  It can be confusing. I’m not sure many understand why we’re here, or what our purpose is. #42  I think it’s confusing for people- I think people get overwhelmed #41  And it’s really overwhelming and it’s really confusing, and the system is very complex for people to have to travel. #41  I think it’s very confusing because in the emergency that there’s so many people that they get – that they see over a few hours in the emergency department and if there’s a change of shift then they have – they have a morning nurse and then they have an afternoon nurse and then they have – they’ll have a different doctor. Yeah. So – and then, yeah – I think it’s just very confusing. And if a person doesn’t have an advocate or a family member there for them. I think the – I think they experience – yeah. Like and if they can’t – if they’re unwell they can’t advocate for themselves. #43  Yep, I will be called by triage if somebody doesn’t necessarily need to be admitted or if they’re becoming distressed, et cetera and I will go out and touch base with them and explain that we are really busy, you are on the list and we’re trying to get you through, is there anything we can do at the moment? That’s my practice anyway, I don’t know if it’s right or wrong practice, but I feel it makes that experience a little bit easier. When they are in the ED waiting, sometimes it is nice to touch base with them, provide family with a chair, introduce who you are, give that familiar face again and explain what’s going on in the emergency department. And that they’ve not been neglected, and they will be seen … if you address those parts, you can make it really smooth but if those bits are neglected, I think some patients can become quite overwhelmed, frustrated, feel as if they’re being neglected, they see doctors and nurses walking around but they don’t realise that those guys and girls have got a thousand other things that are racing through their head. #44 |
| Connections | Managing distress at hospital triage | Yep, I will be called by triage if somebody doesn’t necessarily need to be admitted or if they’re becoming distressed, et cetera and I will go out and touch base with them and explain that we are really busy, you are on the list and we’re trying to get you through, is there anything we can do at the moment? That’s my practice anyway, I don’t know if it’s right or wrong practice, but I feel it makes that experience a little bit easier. When they are in the ED waiting, sometimes it is nice to touch base with them, provide family with a chair, introduce who you are, give that familiar face again and explain what’s going on in the emergency department. And that they’ve not been neglected, and they will be seen. #44  And I think that’s quite distressing for a lot of patients cause there is quite a considerable number that we find the appointments actually been lost or never made or may have been sent to a wrong address. #39 |
| Connections | Immediacy of connection with client (first encounter) | … first phone call, being able to make that in a timely manner. #48  first call and I think trying to clearly explain what you are without overwhelming them. #49  … that importance of that first phone call, being able to make that in a timely manner … that first port-of-call around, I'm the person who's going to be organising this; it's a complex system. #47  So, from that first point, you’ll know how much information you’re actually able to give the person or what information you’re able to give. #40  So, it’s trying to appease various people at different levels, so that first phone call, yep, is really pivotal. #39  I’d say yep, that intake is a critical point to know that the referral has started. That they’re now hooked into the system and that priority is being made and the assurance that – and ascertain how quickly they need to be seen, I’d say that’s a critical point first of all. #9  I think the immediacy. I think that’s really vital and that’s the thing that I really feel passionate about, that we’re - we get the referral usually that day or within 24 hours of the patient going home and where we can make that phone call immediately to the family to say, Look, we’re here, we know that you’ve got a need, we’re trying to help you, how can we assist? Those families respond really positively, and it’s just really been official to be able to do that, it’s just a lot of the families are feeling unsupported, they feel like they’re being pushed out of hospital before their mother was ready or before they were ready to go home. And they feel unloved in lots of ways and just to have someone call them to say, Look, we know this situation is there, we know you’re going to need some shopping or some home cleaning assistance, we’ll work on that, do you want to have a little think about how we could best do that, what days would suit you, what times would suit you? You may not be arranging them exactly there and then but just to know that they’ve got someone to call, a number to call. #39  Well, I think the access at the phone call – the initial phone call is really important, and I think if people have taken so long to find us that they – if they get fobbed off at that point, then I think it’s really bad that that happens. And – so generally I think, most people once they find us that they should really speak to the intake worker, if that’s the problem they’ve got. I think the intake is a crucial part and I think we’ve got really skilled intake clinicians and they do a really good job cause – so, they can – I mean, I’ve done intake over the years too, so you’re part trying to – it’s a real skill, you’ve got to get the information but you’ve also got to feel that that person feels that they’ve been listened to and that you’ve got the key points that are relevant to that referral and then do the leg work. So, I think that’s really important that the person who rings on intake feels that they’ve been listened to – the client – the carer has been listened to. And you might make some suggestions too and then – and half the time the people just feel that they’ve managed to pour out their story to the right person who is then going to get someone to come… #41 |
| **Care planning and flow of treatment** | Client no idea how to navigate system: Client slips through the cracks | I think the intake is a crucial part and I think we’ve got really skilled intake clinicians and they do a really good job cause – so, they can – I mean, I’ve done intake over the years too, so you’re part trying to – it’s a real skill, you’ve got to get the information. #41  Assisting them to be able to communicate with the nursing staff, the doctors and actually navigate through what’s going on in emergency and being their voice. 0#44  So, I think post-acute care does a fairly good job at trying to help people navigate that system initially, but I don't think we're a magic wand that suddenly makes the system [laughs] coordinate and work well. #47  So, knowing how to navigate systems, I think having the courage of your convictions that you are right, you need to help and also then I suppose understanding all the community stuff is how you feel you can advocate best for them. #48  Not many of those fall through the cracks, wound care, look I’m sure we only get a small percentage of the wound care ones that go through; some are sent to GP, some are sent home with family doing it with little understanding of whether the family have actually - well often it’s only been one education session on the ward. You don’t actually know how they’re managing at home, and if they’re not managing, they haven’t been given any contact numbers, it’ll be just ‘go to your GP’ and then it often is depending on your GP as to what happens from that stage. #39  Yeah, it’s easy for people to slip through the cracks. And if people haven't gone home with a letter from the hospital about the plan e.g. referred to outpatients, the GP, post-acute care and the council. If you don't know the plan it's very hard for people to take control. #47 |
| Connections | Being listened to over a period of time | On the positive side, for them, like I said, they’re usually really clear if someone’s actually listening; who’s got the time to sit down for one and a half, two hours at that first visit and get all that information from them. A lot of them, they feel like they’re heard. They’re acknowledged and respected and heard. #33  So, I just feel we’re a vital ongoing link and look, I truly believe that because we get in early, ring early, listen to the patients, a lot of them, they feel they’ve had a bad experience in hospital, listen to them. #39  … I think that’s really important that the person who rings on intake feels that they’ve been listened to – the client – the carer has been listened to. And you might make some suggestions too and then – and half the time the people just feel that they’ve managed to pour out their story to the right person who is then going to get someone to come … #41  It’s hard to explain but I see the role on intake as listening and teasing all that out a bit and – yeah, trying to get the best fit for the client – for the client, the older person and whoever it is that’s making that call. #46  I think – well, the first week or two is not a point, it’s a period of time, but I think if you can be there for them in the first week at home and get things sorted out and give them a sense of some kind of structure so that their overwhelming confusion feeling can be managed so you might have to impose a structure on them such as getting a folder with clear plastic things in it that they can put letters in and recommending an exercise book to write things down, phone calls and messages. Really basic things like that. #49 |

| **Table C Selected verbatim quotes: where clinicians perceived services had failed, and what they perceived could be done to improve** | | |
| --- | --- | --- |
| Picker theme | Where clinicians perceived services had failed | What clinicians perceived could be done to improve |
| Connection, Consistency, Information and knowledge | **Discharge planning (fragmented discharge planning**)  In general, most of our clients don't have that [laughs] information [written discharge plan] and aren't very assertive. That's the word I was looking for. They're not very assertive I think, that's the nature of people in the western suburbs. The hospital said they’ve organised something and the client just waits. #47  **Referrals lack quality due to stretched hospital systems**  … think having worked in post-acute care on and off for about 10 years, I'm aware how much more this system, both the hospital system and the external system is stretched and stressed. And so, the quality of the referrals and the ability of people on the wards to provide quality referrals is limited. #47 | **Improved discharge follow-up**  Being able to like follow-up on patients, even just doing a phone call to – if they get discharged, like do a telephone call, follow up to see that they’re managing when they go home or if there’s any concerns, and doing an outreach visit. #43  **Better explanations to clients on discharge**  I would like to see an improvement around explanations to clients on discharge. #47  **Poor referrals frustrating and more time needs to be spent delving into medical record, enhancing education of clinical staff and streamlining paperwork**  I think the poor referrals, I think that that frustrates staff a lot, I think it’s something that we’ve just got to adjust to and we’ve just got to be prepared to spend more time looking into the medical record and looking up patient needs and using judgement calls. I can’t see that that’s going to improve, and I mean we can look at doing education and we can look at all those other factors, but I think it’s just the lack of time that staff on the ward have got to fill in paperwork. I think it is probably confusing for staff at the moment, I don’t think we’ve educated particularly well, and we are working on that. #39  **Better networking with problematic wards**  And networking and we'll suddenly have a situation where a particular ward is very difficult to deal with and provides quite poor-quality referrals. #47  **Need to ask questions yourself to enhance often-delayed referral information**  Cause that information is not so clear and the reason that is often is like I said before, we often get referrals that are just the day before and you’re going in there blindly trying to put this person on because they’re being discharged that day. So, you can’t get all the information that you ideally want and then you clearly noted to your colleagues in the hospital that you need this information, please forward it on and it doesn’t come all at once. It comes in dribs and drabs over a couple of weeks’ time. And by then, you’re well into it; you’ve already discovered what you needed to discover by asking the questions yourself. #42 |
| Connection | **Lack of interdisciplinary connections related to ‘churn’ of patient and stretched hospital system**  There’s difficulties that are increasing in the role because of the speed and numbers that patients are going through the ward, the lack of information or the poor information that we’re getting about the client. Often, we’re getting incorrect information about the client, clearly in a lot of cases, all the needs of the clients aren’t being considered so they’re referred by a single discipline. So, the physio refers them, she hasn’t actually spoken to the OT or the nursing staff to find out where there are the needs that were required for this. #39 | **Active management on part of clinician re caseload**  …some of that active management around what's a reasonable case load and what's a reasonable level of follow up and what's too much follow up and what's a bit slap-dash! #47  **Follow up with social workers key to ensure appropriate discharge planning**  One of the biggest difficulties I do find is when we’ve spent so much time working with someone, put the referrals through to the ward, they tend to get lost in the rest of the hospital. And you have them re-present again and you say: ‘Did you see the social worker, did you see this person?’ And no-one’s fault but because it’s really focused on discharge planning. Those social workers, physios, OTs don’t get the time to really put in and follow up. So, they’re lost where they’re pushed through and then out home, and then they re-present with the same scenario; you put that hard work in again and I have actually had it where I’ve gone up to the ward, just to follow up, to make sure that social workers are involved. #44  **Education about care coordination role**  Education, I think of our role, whether it be with other care co-ordinators or other staff just so then they understand why we need to know what we do, what we do and, again, chasing referrals that can be the most frustrating, chasing detail and then at times, often a hostile reaction… #48 |
| Consistency |  | **Clear communication about the role and purpose of the service with internal clinicians**  Clear information about who the team members are involved in that client’s care. How often they’re going to be seen and how they can be contacted would probably make a difference. #42  **Clear communication of care expectations to client on discharge**  I guess that the communication, what I was saying before, when they leave here that they’re clear on what’s going to happen. And we have sort of – well, one of the other care coordinators came up a letter that we give them, and we can summarise what we’re organising for them. #45 |
| Collaboration and Fragmentation of holistic care | **Many fingers in the pie and confusing for patients. Poor discharge planning leads to client confusion.**  … because we are a remote model, effectively we have no control over what the client is told about whether they've even been referred to post-acute care even though they've technically given consent or what message they're given. So, we're basically calling them, usually after discharge to say hello it's Sally (pseudonym) from post-acute care at Sunshine Hospital and explaining. And sometimes they'll say the ward talked to me about that and that you're going to provide some short-term physio and you're funding the nursing. And other times people will say no, they didn't discuss this with me. So, you're saying here's the service, here's what we provide; here's why the ward thought that you might need some assistance with this. So yeah, the amount of information the clients have is very fluctuating, if that makes sense. So, I think it's quite confusing getting a call out of the blue depending on what's been explained. I think the other thing is when you first go home from hospital, you often get a called fairly frequently by a range of services. Often council will give them a call as well as RDNS and PAC. They might have to ring their pharmacist about something. So, there might be five or six people all putting a finger in the pie fairly quickly after discharge. So, I think that's fairly confusing. Hopefully, at the end of the PAC phone call, they're going that's great, thank you very much. That's now a lot clearer. But I do think a fair percentage of the time when we ring people; they're not really clear on what the plan was. #47 | **Information about client packages needs to be communicated clearly by clinicians**  The confusion over the packages, a lot of people don’t understand what the packages are. And then there’s all those problems within that, so if you ring people and say you’ve been waitlisted for a package, they don’t understand what a package is, so… #41  **Instills confidence in consumer that clinicians are abreast of their care needs and care planning coordinated**  Look, I got this information from my colleague in the emergency department, she said that you have got, boom, boom, boom, is that all correct? So again, exactly, the client feels more confident that you have actually got that, you’ve got that information, that’s all been transferred, they also don’t get fed up with repeating themselves. #46  …but your gut feeling by their verbalisation and by their reaction is that yes, they needed someone to talk to, they needed that confidence, they needed the thought that someone was going to look after them if they stayed at home, yeah. #39  A carer might ring because they’re worried about leaving mum or dad alone and they’re about to go on two weeks holiday overseas and the carer might make a fairly straight forward phone call to us saying, I need to organise the official paperwork to get respite organised, in a facility because I can’t leave mum or dad alone for the two weeks I’ll be away. #46 |
| Collaboration and Fragmentation of holistic care | **Families feel unsupported because they perceive discharge from hospital is too early**  It’s just a lot of the families are feeling unsupported, they feel like they’re being pushed out of hospital before their mother was ready or before they were ready to go home. #39  So, in that part of my work, I try and stop wards from discharging people who will a) be unsafe at home and b) be unsupported and c) go home without things in place that it’s the ward’s job to put in place, e.g. nursing support, medication and things like that. #49 | **Letting families that feel ‘unloved’ know there is number and person to call**  And they feel unloved in lots of ways and just to have someone call them to say: ‘Look, we know this situation is there, we know you’re going to need some shopping or some home cleaning assistance, we’ll work on that, do you want to have a little think about how we could best do that, what days would suit you, what times would suit you?’ You may not be arranging them exactly there and then but just to know that they’ve got someone to call, a number to call. #39  And we will occasionally get phone calls from people months down the track and that’s fine, sometimes it’s nice to hear how they’re going because often with people you’ve built up a bit of a rapport with: ‘So, I’ve got your number written down, Brigid (pseudonym), you’ll help me?’ #48 |
| Connection | **Lack of culturally diverse staff**  I think within our cohort, we probably struggle to pick up certain CALD backgrounds. I think that’s cultural. Particularly, for example, the Vietnamese population. I don’t think we do as well with them as we potentially could. They’re very difficult to engage. #2 | **Vietnamese speaking care facilitator needed to cater for high numbers of Vietnamese speaking consumers**  I think if we had a Vietnamese speaking care facilitator, it would make a huge difference. #2 |
| Care Planning & Flow of Treatment | **Doubling up and wasting time**  We are swamped with double paperwork, double computer entry. #33  … if we didn’t have to do double [laughter] data entry, we’d have more time. #40  You can't be spending this much time doing this wonderful work if somebody else is doing double desks for three days and you're saying you don't have capacity. We've got to even it out and I'm aware of that basically. #47 | **Communication reduces doubling up on work activity**  Because we all work better together, we all talk to each other more so there’s less doubling up for them. #32  **Co-location offered as an approach to help with doubling up**  … often think that community services such as PAC TCP IRS HARP double up a lot …And I think a lot of time is spent doubling up and consequently wasting time. We find out that – doubling up not necessarily on services but doubling up on information and I’ve always had this idea that if we were all co-located to a certain degree, we could work a lot more effectively. #48 |
| Fragmentation of Holistic Care | **Clients can suffer loss of control, powerlessness, if decisions made on the run for them**  I don’t think I can answer in a general way expect maybe there are some issues that are the same across a lot of people that would be powerlessness, helplessness, very often confusion about who’s what, where, what's happening, who are they, and so on, intrusion into privacy, that the home could feel like it’s become Bourke Street with people coming in and out every day. For some people who are very private, that's a real ordeal. A sense of disempowerment that other people are making decisions for you and organising things for you. #49 |  |
